# Acquired HIV-1 Protease Conformational Flexibility Associated with Lopinavir Failure May Shape the Outcome of Darunavir Therapy after Antiretroviral Therapy Switch

**DOI:** 10.3390/biom11040489

**Published:** 2021-03-24

**Authors:** Simeon Eche, Ajit Kumar, Nelson Sonela, Michelle L. Gordon

**Affiliations:** 1 Discipline of Virology, School of Laboratory Medicine and Medical Sciences, University of KwaZulu-Natal, Durban 4001, South Africa; echesimeon@gmail.com; 2 Discipline of Microbiology, School of Life Sciences, University of KwaZulu-Natal (Westville Campus), Durban 4000, South Africa; ajitkanwal@yahoo.com; 3 School of Medicine, Physical and Natural Sciences, University of Rome Tor Vegata, 1-00133 Rome, Italy; nelsonela@yahoo.fr; 4 Chantal Biya International Reference Center for Research on the Management and Prevention of HIV/AIDS (CIRCB), Yaoundé P.O. Box 3077, Cameroon

**Keywords:** HIV-1 protease, HIV-1 protease inhibitor, lopinavir, darunavir, conformational flexibility

## Abstract

Understanding the underlying molecular interaction during a therapy switch from lopinavir (LPV) to darunavir (DRV) is essential to achieve long-term virological suppression. We investigated the kinetic and structural characteristics of multidrug-resistant South African HIV-1 subtype C protease (HIV-1 PR) during therapy switch from LPV to DRV using enzyme activity and inhibition assay, fluorescence spectroscopy, and molecular dynamic simulation. The HIV-1 protease variants were from clinical isolates with a combination of drug resistance mutations; MUT-1 (M46I, I54V, V82A, and L10F), MUT-2 (M46I, I54V, L76V, V82A, L10F, and L33F), and MUT-3 (M46I, I54V, L76V, V82A, L90M, and F53L). Enzyme kinetics analysis shows an association between increased relative resistance to LPV and DRV with the progressive decrease in the mutant HIV-1 PR variants’ catalytic efficiency. A direct relationship between high-level resistance to LPV and intermediate resistance to DRV with intrinsic changes in the three-dimensional structure of the mutant HIV-1 PR as a function of the multidrug-resistance mutation was observed. In silico analysis attributed these structural adjustments to the multidrug-resistance mutations affecting the LPV and DRV binding landscape. Though DRV showed superiority to LPV, as a lower concentration was needed to inhibit the HIV-1 PR variants, the inherent structural changes resulting from mutations selected during LPV therapy may dynamically shape the DRV treatment outcome after the therapy switch.

## 1. Introduction

Globally, HIV-1 infection remains a serious public health problem, with about 38 million infected people at the end of 2019 [1]. The global HIV-1 epidemic burden rests heavily on countries in sub-Saharan Africa [2]. South Africa remains the global epicenter of the HIV-1 epidemic, with the pandemic dominated by the HIV-1 subtype C [3,4]. The standard treatment of HIV-1 infection is highly active antiretroviral therapy (HAART) [5]; HAART has greatly improved the clinical outcome of HIV-infected persons since its introduction [6]. However, the emergence of drug-resistant HIV-1 variants has significantly contributed to the failure to control HIV-1 replication in some patients [6]. The increasing cases of virological failure associated with first and second-line antiretroviral therapy (ART) present a significant clinical challenge for patient management in resource-constrained settings [7]. The ability to provide effective and sustained virological suppression using ART is crucial; thus, HIV-1 protease inhibitors (PIs) with a high genetic barrier to the evolution of drug resistance forms the second and last-line ART in many settings globally [6,7].

**Figure 1:**
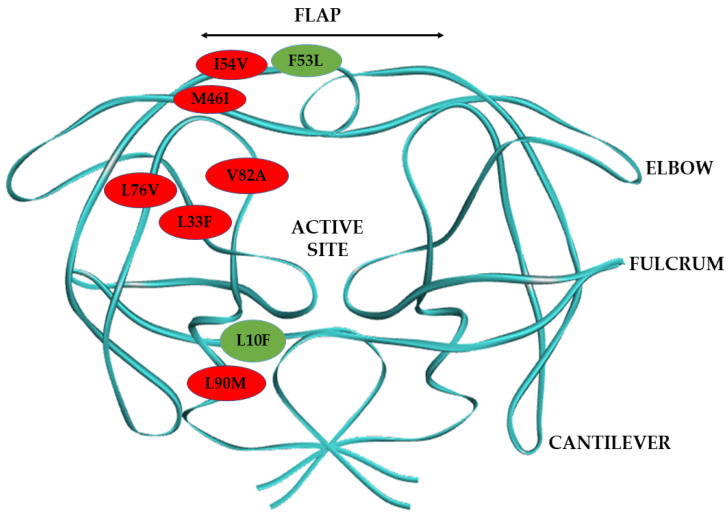
Diagrammatic representation of HIV-1 PR dimer structure and the mutations harbored by the mutant HIV-1 PR variants in this study. The major HIV-1 PR mutations are represented in red balls and the minor mutations in green balls.

HIV-1 protease (PR) is a key drug target that plays a crucial role in cleaving newly synthesized viral polyprotein into functional proteins needed for the maturation of nascent viral particles [8]. The HIV-1 PR is a 99 amino acid homodimer, organized into six structural segments (Figure 1), namely: the flap region, made up of residues 43–58/43′–58′; the flap elbow, consisting of residues 35–42/35′-42′; the fulcrum, which comprises of residues 11–22/11′–22′; the cantilever, which is made up of residues 59–75/59′–75′; the dimer interface, made up of residues 1-5/1′-5′, 95-99/95′-99′; and the catalytic site, which comprises residues 23–30/23′–30′ [9,10]. The flap covers the HIV-1 PR active site, and it regulates the entry of substrates and HIV-1 protease inhibitors (PIs) into the catalytic site [11]. HIV-1 PIs are non-cleavable substrate analogs designed to bind to the active site of HIV-1 PR. The binding of HIV-1 PIs in the enzyme active site inhibits its normal enzymatic activity by preventing it from cleaving its natural substrate [12]. However, the antiviral capacity of HIV-1 PIs and the affinity of HIV-1 PR for these inhibitors is diminished by drug resistance mutations in the HIV-1 PR gene [13,14].

Drug-resistance mutations could be found in the active site of HIV-1 PR and directly impact the binding affinity and interaction of HIV-1 PIs with HIV-1 PR [15]. In contrast, non-active site mutations may not directly affect the interaction of HIV-1 PR with inhibitors but may indirectly influence the molecular interaction of inhibitors with the HIV-1 PR through alteration of the protein flexibility and stability [16,17,18]. The accumulation and interplay between active and non-active site drug-resistance mutations arising from drug pressure may cause structural changes, leading to HIV-1 PR variants with altered protein conformations [19,20]. The alteration in the conformations of HIV-1 PR confers on it adaptability and flexibility, thus affecting its interaction with different HIV-1 PIs [21,22]. The gain in HIV-1 PR flexibility due to changes in inter-residue connections alter the HIV-1 PI binding landscape, resulting in the inability of HIV-1 PIs to bind firmly in the active site [23].

In low and middle-income countries, boosted lopinavir (LPV) has been the common backbone of the second-line ART regimen [24]. The combination of ritonavir-boosted lopinavir (LPV/r) with nucleoside reverse transcriptase inhibitors (NRTIs) has been a well-utilized, cost-effective treatment regimen for the management of HIV-1 infection [25]. However, the development of resistance to an LPV-based regimen affects its use in patient management. Its continuous use during virological failure may result in significant cross-resistance to other HIV-1 PIs [24]. The major HIV-1 PI resistance mutations that affect the efficacy of boosted LPV regimen are V32I, L33F, M46I/L, I47V/A, I50V, I54V/T/A/L/M, L76V, V82A/F/T/S, I84V. and L90M [26]. Research shows that the emergence of V32I, L33F, I47A, I50V, L76V, and 184V under drug pressure during LPV therapy may confer cross-resistance to DRV [27,28]. Where LPV treatment fails, DRV is an effective salvage remedy [29] because of its high genetic barrier to drug resistance, making it the preferred antiviral agent used in numerous HIV-1 treatment plans for therapy-naive and experienced patients [30]. In addition, DRV’s dual mechanism of action by directly inhibiting HIV-1 PR, and inhibiting HIV-1 PR dimerization, puts it in a better position as the HIV-1 PI of choice for salvage therapy [6].

Currently, there is no comparative analysis of the biochemical and structural implications of this regimen switch in patients harboring multidrug-resistant South African HIV-1 subtype C PR. This study aims to gain mechanistic insight into the development of resistance to LPV by drug-resistant HIV-1 subtype C clinical isolates at the point of regimen switch to DRV, using biochemical and in-silico analysis. This study also seeks to determine how well DRV would fare in managing patients failing LPV-based therapy when their treatment regimen is changed. The South African HIV-1 subtype C PR variants studied harbored major and minor HIV-1 PI drug-resistant mutations (Figure 1). The major PI mutations are M46I and I54V found in the flap region, L76V located in the central β-sheet that forms one of the active site’s borders, the V82A mutation, which is found in the active site; and the L90M and L33F mutations in the HIV-1 PR hydrophobic core. The minor drug resistance mutations are L10F located around the fulcrum, and the F53L—a flap mutation. Research has shown that the most reported major HIV-1 PI resistance mutations in patients failing second-line therapy in South Africa are I54V, V82A, and M46I [31,32]. Therefore, in this study, we chose mutant HIV-1 PR variants harboring different combinations of the common HIV-1 PI resistance mutations reported in the South African HIV-1 subtype C. This study will improve the available knowledge to develop more efficient HIV-1 PIs considering the unique characteristics of multidrug-resistant South African HIV-1 subtype C PR.

## 2. Materials and Methods

### 2.1. Ethical Approval

This study was approved by the University of KwaZulu-Natal Biomedical Research Ethics Committee (BREC NO. 413/17).

### 2.2. Amplification of HIV-1 Protease 

Blood samples were obtained from consenting patients failing second-line lopinavir (LPV) inclusive regimen and switching to darunavir (DRV) treatment from an observational cohort study in Durban, South Africa. Reverse transcription polymerase chain reaction (RT-PCR) and nested PCR were used to amplify HIV-1 gag-protease region from plasma RNA extracted using the QiAmp Viral RNA Kit (Qiagen, Germantown, USA). RT-PCR and nested PCR conducted as described previously [33].

Briefly, the Superscript III One-Step RT-PCR System with Platinum Taq DNA Polymerase (Invitrogen, Carlsbad, CA, USA) was used to perform the RT-PCR using HIV-1 gag—protease specific primers; Gag+1: 5′-GAGGAGATCTCTCGACGCAGGAC-3′ as the forward primer and 3′rvp: 5′_GGAGTGTTATATGGATTTTCAGGCCCAATT_3′ as the reverse primer. The PCR conditions were an initial of 55 °C for 30 min to generate cDNA, followed by a denaturation cycle at 94 °C for 2 min, 35 cycles at 94 °C for 15 s, 55 °C for 30 s and 68 °C for 2 min and final extension at 68 °C for 5 min. Using the first round PCR products as template, nested PCR was carried out using TaKaRa Ex Taq HS enzyme kit (Takara, Shiga, Japan) with the following primers Long_fwd: 5′_GACTCGGCTTGCTGAAGCGCGCACGGCAAGAGGCGAGGGGCGGCGACTGGTGAGTACGCCAAAAATTTTGACTAGCGGAGGCTAGAAGGAGAGAGATGGG_3′ and Long_rev: 5′_GGCCCAATTTTTGAAATTTTTCCTTCCTTTTCCATTTCTGTACAAATTTCTACTAATGCTTTTATTTTTTCTTCTGTCAATGGCCATTGTTTAACTTTTG_3′ under the following PCR conditions: an initial denaturation at 94 °C for 2 min then 40 cycles at 94 °C for 30 s, 60 °C for 30 s and 72 °C for 2 min), with a final extension at 72 °C for 7 min.

HIV-1 PR gene was then amplified from the HIV-1 gag-protease PCR product using the primers pMal_Fwd: 5′_CAGCGGCCGCGGAGAAGAAAGACAGGGAACC_3′ with a Not1 restriction site attached to the 5′ and MdfINPR_Rev: 5′_TACGAATTCCCTGGCTTTAATTTTACTGGTACAG_3′ with an EcoR1 restriction site at the 5′ end using the following PCR conditions: an initial denaturation cycle at 94 °C for 2 min, followed by 30 cycles at 94 °C for 15 s, 58 °C for 30 s and 72 °C for 45 s, and a final cycle of extension (72 °C for 7 min). The amplified products were bulked sequenced using the ABI Prism Big Dye Terminator v3.1 Cycle Sequencing Kit (Applied Biosystems, San Francisco, CA, USA).

### 2.3. Cloning, Expression and Purification of HIV-1 Protease

HIV-1 PR PCR product and pMAL-c5X expression vector (New England BioLabs, Ipswich, MA, USA) were both digested using FastDigest restriction enzymes Not1 and EcoR1 (Thermo Scientific, Waltham, MA, USA) and ligated into the pMAL-c5X expression vector according to the manufacturer’s instructions. The ligation mixture was transformed to One-shot Top10 competent cells (Invitrogen, Carlsbad, CA, USA), plated, and incubated in Luria Bertani (LB) ampicillin (100 μg/mL) agar plate overnight at 37 °C. The presence of HIV-1 PR in clones was confirmed using colony PCR, and the positive clones were bulked sequenced using the ABI Prism Big Dye Terminator v3.1 Cycle Sequencing Kit. Plasmid DNA isolated from a single positive clone was then used to transform NEBExpress *Escherichia coli* cells (New England BioLabs, Ipswich, MA, USA), cultured on ampicillin agar plates, and incubated overnight at 37 °C. The colonies were screened for positive clones using PCR and used for HIV-1 PR expression.

To express and purify HIV-1 PR, a single positive clone was inoculated overnight in LB ampicillin (100 μg/mL) media at 37 °C and shaking at 230 rpm. Of the overnight culture 10 mL was inoculated to l liter of LB ampicillin media containing glucose (0.2%) and then induced with 0.3 mM IPTG after 4 h (OD600 = 0.5). The cells were harvested after 3 h by centrifuging at 4000 × *g* for 20 min and resuspended in 25 mL of buffer A (20 mM Tris-HCl, pH 7.4, 200 mM NaCl, 1 mM EDTA, 1 mM DTT, and 1 mM azide). The cells were sonicated (CML-4, Thermo Fisher, CA, USA) in short pulses of 15 s in an ice-water bath, and the supernatant containing the HIV-1 PR fusion protein was collected by centrifugation at 20,000× *g* for 20 min. The MBP tagged HIV-1 PR was then purified using the 5 mL MBP Trap HP column (GE Healthcare, Piscataway, NJ, USA) according to the manufacturer’s protocol. The MBP tag was cleaved from the fusion by treating it with factor Xa (New England BioLabs, Ipswich, MA, USA) followed by dialysis with buffer B (20 mM Tris-HCl and 25 mM NaCl, pH 8.0) and using the HiTrap Q FF column (GE Healthcare, Piscataway, NJ, USA). Factor Xa cleavage protease was removed using the HiTrap Benzamidine column (GE Healthcare, Piscataway, NJ, USA). The expressed HIV-1 PR samples were folded by diluting 10-fold with buffer C (0.05 M Na-acetate, 5% ethylene glycol, 10% glycerol, and 5 mM DTT, pH 5.5) [34]. For every experiment, fresh protein samples were refolded. The protein expression and purity were checked by SDS-PAGE [35] at every step, and the concentration of the protein obtained using absorbance spectroscopy at 280 nm. The free HIV-1 PR protein was further confirmed using a Western blot.

### 2.4. Western Blotting to Detect the Presence of HIV-1 PR

The purified HIV-1 samples (200 ng) were loaded into the wells of the SDS-PAGE gel and allowed to run at 110 V for 45 min. After SDS electrophoresis, the protein bands after SDS electrophoresis were transferred to the nitrocellulose membrane using the Trans-Blot Turbo Transfer System (Bio-Rad, Hercules, CA, USA). The nitrocellulose membrane was blocked in 5% BSA (containing 0.1% Tween 20) for 2 h on a shaker. The 5% BSA was discarded, and a 1:1000 dilution of the primary antibody (Anti-HIV protease, EXBIO Praha, Vestec, Czech Republic) was added to the nitrocellulose membrane, left on the shaker for 1 h, and then stored overnight at 4 °C. The nitrocellulose membrane was then subsequently washed 5 times in wash buffer (10× Tris-Buffered Saline (TBS), and a 1:1000 dilution of the secondary antibody (Anti-Human IgG H&L, HRP, Abcam, United Kingdom)) was added and placed on the shaker for 2 h. The nitrocellulose membrane was then washed a second time. The Pierce ECL Western Blotting Substrate (Thermo Fisher Scientific, MA, USA) was added to the membrane and visualized in a light-sensitive film. 

### 2.5. Enzyme Activity Assay and Inhibition Studies

HIV-1 PR activity was measured by adding a purified enzyme (100–300 nM) to 300 µM of the chromogenic substrate Lys-Ala-Arg-Val-Nle-p-nitro-Phe-Glu-Ala-Nle amide (Sigma-Aldrich, St. Louis, MO, USA) dissolved in buffer D (50 mM sodium acetate buffer containing 100 mM NaCl (pH 5.0)) at 37 °C. The change in absorbance upon hydrolysis of the substrate by HIV-1 PR was monitored, and an extinction coefficient of 1800 M-1 cm-1 at a wavelength of 300 nm was used to convert the absorbance change to reaction rates [36,37]. The inhibition of HIV-1 PR activity by the inhibitors was studied by measuring the enzyme activity in the presence of 0–500 nM of LPV and DRV, using three substrate concentrations: 100, 200, and 300 μM respectively, in buffer D at 37 °C. The data obtained were analyzed by plotting the reciprocal of substrate hydrolysis against substrate concentration (Lineweaver–Burk method [38] to determine the *K*_m_ and *K*_cat_) and inhibitor concentration (Dixon method [39] to determine the *K*_i_) respectively. The relative resistance was calculated by dividing the *K*_i_ value of the mutant HIV-1 PR variants by the *K*_i_ value of the wild type for LPV and DRV respectively.

### 2.6. Fluorescent Spectroscopy

Fluorescent spectra were recorded as described previously [40] using a PerkinElmer LS 55 spectrometer with a 1.0 cm quartz cell (PerkinElmer, Waltham, MA, USA) and connected to a thermostat-controlled bath. This assay utilized the intrinsic tryptophan (Trp) fluorescence by causing the excitation of the π–π* transition in the tryptophan residues. Two Trp residues are found at positions 6 and 42 in each monomer of HIV-1 PR. Trp 6 is located close to the active site, and Trp 42 is found close to the flap of the HIV-1 PR. The position of the Trp residues on the enzyme surface makes them good probes in monitoring changes in the HIV-1 PR tertiary structure [40]. The protein was excited at 295 nm, and fluorescence measurements were recorded from 300 to 420 nm at room temperature (25 °C). The excitation and emission slit widths were set at 5 nm, and the fluorescence spectra were acquired at 500 nm/min. The obtained fluorescence data was corrected and smoothened by running control samples of the buffer. For the fluorescence quenching study, 300 nm of HIV-1 PR and varying inhibitor concentration from 10 to 500 nM were used. For every reaction, a new enzyme solution was used. The decrease in intrinsic Trp fluorescence (F_0_–F) at each concentration of inhibitor was fitted to the equation (F_0_–F) = ΔF_max_/(1 + (*K*_i_/[I]) to determine *K*_i_ and ΔF_max_ values using the Origin(Pro), 2019 (OriginLab Corporation, Northampton, MA, USA). The Stern–Volmer quenching constants (*K*_sv_) were also calculated by fitting the data into the equation F_0_/F = 1 + *K*_sv_ (Q). The Stern–Volmer constant reports the accessibility of fluorophores to a quencher and the solvent accessibility of the fluorophore. Thus, it is an essential tool that can be used to probe the conformational changes around a fluorophore in proteins [41]. It is also an indication of the inhibitors’ quenching capacity, the higher the *K*_sv_ value, the greater the quenching. The inner filter effect was corrected by using the formula F_c_ = F_antilog_[(A_ex_ + A_em_)/2], where F_c_ is the corrected measurement and F is the measured fluorescence intensities, respectively, A_ex_ is solution absorbance at the excitation, and A_em_ emission wavelengths [42]. 

### 2.7. Computational Methods

#### 2.7.1. HIV-1 PR Enzyme and HIV-1 PIs System Preparation and Molecular Docking 

The monomeric form of wild type (WT) South African HIV-1 subtype C PR X-ray crystal structure (3U71) was retrieved from the RSCB Protein Data Bank [43] and converted to a dimeric structure using the UCSF Chimera software [44]. The mutant South African HIV-1 PR (MUT-1, MUT-2, and MUT-3) structures were obtained through homology modeling performed on the SWISS-MODEL web server. The wild type South African HIV-1 subtype C PR x-ray crystal structure (3U71) as a template. The structure of FDA-approved protease inhibitors (PIs) DRV and LPV were obtained from PubChem [45], and the Avogadro software package was used to prepare the 3-D structures of the PIs [46]. Molecular docking was utilized to predict the ligands’ best geometric conformation within the HIV-1 PR active site. The Autodock Vina Plugin available on Chimera software was used for molecular docking [44], with default docking parameters. Prior to molecular docking, Gasteiger charges were added to the HIV-1 PIs; DRV and LPV, also the non-polar hydrogen atoms, were merged to carbon atoms. The HIV-1 PIs were then docked into the HIV-1 PR binding pocket and subsequently subjected to molecular dynamic (MD) simulations.

#### 2.7.2. Molecular Dynamic (MD) Simulations

MD simulations were performed using the GPU version provided with the AMBER 18 package. The AMBER 18 package Leap module was used for the addition of Na+ and Cl- ions to neutralize the system. Atomic partial charges for the ligand were generated using ANTECHAMBER, by utilizing the restrained electrostatic potential (RESP) and the general amber force field (GAFF) procedures. The systems were described using the AMBER ff18SB force field parameters [47]. Amino acid residues of the proteins were renumbered based on the dimeric form of the enzyme from 1 to 198. All the systems were suspended implicitly within an orthorhombic box of TIP3P water molecules in such a manner that all atoms were within 8 Å of any box edge. Initial minimization of 2000 steps with an applied restraint potential of 500 kcal/mol for both solutes was carried out. This was performed for 1000 steps using the steepest descent method and then followed by 1000 steps of conjugate gradients. In addition, full minimization of 1000 steps was further performed by the conjugate gradient algorithm without restraint. MD simulation was performed with gradual heating from 0 to 300 K, executed for 50 ps, such that the systems maintained a fixed number of atoms and fixed volume as previously described [48]. MD simulations were performed for 700 ns. Post dynamic analysis was done using CPPTRAJ modules implemented in Amber18 for analysis of the root mean square fluctuation (RMSF), root mean square deviation (RMSD), solvent accessible surface area (SASA), and radius of gyration (ROG) as described by [49]. The active site to flap tip distances (Cα D25–I50 (chain A) and Cα D25′–I50′ (chain B)) and the chain A flap tip to chain B flap tip (Cα I50–I50′) distances for the wild type (WT), and mutant HIV-1 PRs bound to DRV and LPV was explored. These distances are often used to determine the vertical and horizontal movement of the HIV-1 PR flap [50,51]. The Origin Pro, 6.0 (OriginLab Corp, Northampton, MA, USA) data analysis software was used to plot all the graphs [52]. 

#### 2.7.3. Binding Free Energy Calculations

The binding free energies of the systems were determined using the molecular mechanics/generalized born surface area (MM/GBSA) method [53]. Binding free energy was averaged over 100,000 snapshots extracted from the last 100 ns trajectory. The free binding energy (ΔG) was computed for different molecular species (complex, ligand, and receptor) as described by the equations below [54]:

(1) $$\Delta G_{\mathrm{bind}}=G_{\mathrm{complex}}-G_{\mathrm{receptor}}-G_{\mathrm{ligand}}$$

(2) $$\Delta G_{\mathrm{bind}}=E_{\mathrm{gas}}+G_{\mathrm{sol}}-\mathrm{TS}$$

(3) $$E_{\mathrm{gas}}=E_{\mathrm{int}}+E_{\mathrm{vdw}}+E_{\mathrm{ele}}$$

(4) $$G_{\mathrm{sol}}=G_{\mathrm{GB}}+G_{\mathrm{SA}}$$

(5) $$G_{\mathrm{SA}}=\gamma \mathrm{SASA}\;$$

where E_gas_ is the gas-phase energy and encompasses the internal energy E_int_; Coulomb energy E_ele_ and the van der Waals energies E_vdw_. The E_gas_ was determined directly from the FF14SB force field terms. The solvation free energy, G_sol_, is a combination of the energy contribution from the polar states, G_GB_, and the non-polar states, G. The non-polar solvation energy G_SA_ was determined from the solvent-accessible surface area (SASA), using a water probe radius of 1.4 Å, while the polar solvation, G_GB_, was calculated using the GB equation. S and T respectively indicate the total entropy of the solute and temperature.

## 3. Results

### 3.1. Cloning and Expression of HIV-1 PR 

**Figure 2:**
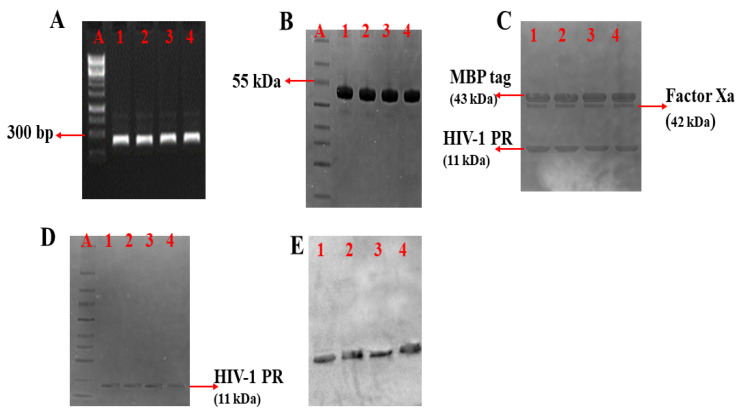
PCR amplification, expression, and purification of HIV-1 PR. (**A**) Amplified HIV-1 PR gene; DNA marker (Lane A), amplified HIV-1 PR wild type (WT) (Lane 1), amplified mutant HIV-1 PR variants (Lane 2 = MUT-1, Lane 3 = MUT-2, and Lane 4 = MUT-3). (**B**) SDS-PAGE showing purified MBP tagged HIV-1 PR variants: Lane A (protein marker), Lane 1 (WT), Lane 2 (MUT-1), Lane 3 (MUT-2), and Lane 4 (MUT-3). (**C**) SDS-PAGE showing cleavage products after Factor Xa cleavage of HIV-1 PR from the MBP tag: Lane 1 (WT), Lane 2 (MUT-1), Lane 3 (MUT-2), and Lane 4 (MUT-3). (**D**) SDS-PAGE showing purified HIV-1 PR variants: Lane A (protein marker), Lane 1 (WT), Lane 2 (MUT-1), Lane 3 (MUT-2), and Lane 4 (MUT-3). (**E**) Western blot picture for the HIV-1 PR variants, Lane 1 (WT), Lane 2 (MUT-1), Lane 3 (MUT-2), and Lane 4 (MUT-3).

The amplified HIV-1 PR PCR product was visualized using agarose (1%) gel electrophoresis (Figure 2A and Figure S1) and confirmed to be approximately 297 base pairs (bp). The wild type and mutant HIV-1 PR genes were successfully cloned into the pMAL expression vector fused to the MBP tag. Colony PCR and Sanger sequencing results confirmed the amplified HIV-1 PR gene and recombinant colonies containing the variants of HIV-1 PR. Furthermore, the HIV-1 PR sequences were uploaded onto the HIV drug resistance database [55] for confirmation of the mutations harbored. The expressed and purified MBP tagged HIV-1 PR had a molecular weight of approximately 55 kDa (Figure 2B and Figure S2). After the fusion tag’s cleavage (Figure 2C and Figure S3), the free HIV-1 PR had an approximate molecular weight of 11 kDa (Figure 2D and Figure S2). The Western blot confirmation of the expressed HIV-1 PR is shown in Figure 2E (full blot picture in Figure S4).

### 3.2. Enzyme Activity of Wild Type HIV-1 PR and Mutants

**Figure 3:**
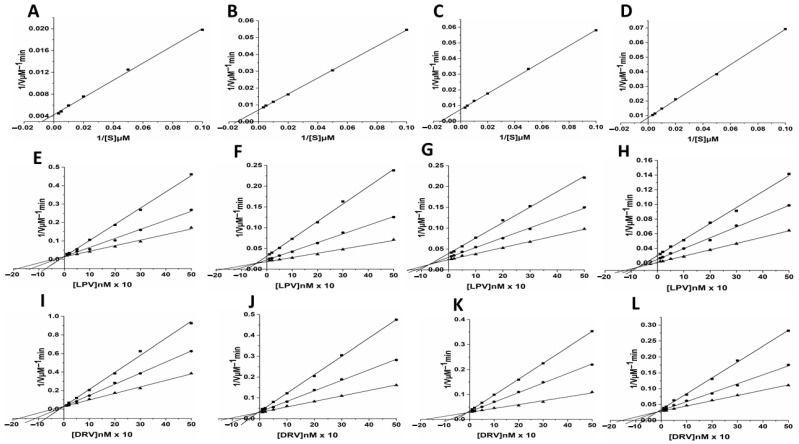
(**A**–**D**) Enzyme kinetic activity of WT (**A**) and mutant HIV-1 PR variants (**B** = MUT-1, **C** = MUT-2, and **D** = MUT-3) was determined following the hydrolysis of the chromogenic synthetic substrate (Lys-Ala-Arg-Val-Nle-p-nitro-Phe-Glu-Ala-Nle amide). (**E**–**H**) The activity of the HIV-1 PR variants (**E** = WT, **F** = MUT-1, **G** = MUT-2, and **H** = MUT-3) measured in the presence of 10–500 nM lopinavir (LPV) using three substrate concentration: 100 (■), 200 (●), and 300 μM (▲) respectively. (**I**–**L**) The activity of the HIV-1 PR variants (**I** = WT, **J** = MUT-1, **K** = MUT-2, and **L** = MUT-3) measured in the presence of 10–500 nM darunavir (DRV) using three substrate concentration: 100 (■), 200 (●), and 300 μM (▲) respectively.

The enzyme activity assay results obtained from the hydrolysis of the chromogenic substrate showed that the wild type (WT) HIV-1 PR had about a 2-fold higher (*K*_m_ = 37.49 ± 0.63 µM) affinity than the mutant PRs (*K*_m_ for MUT-1 = 67.78 ± 1.22 µM, *K*_m_ for MUT-2 = 67.46 ± 1.48 μM, and *K*_m_ for MUT-3 = 70.59 ± 1.01 µM) for the chromogenic substrate (Table 1, Figure 3A–D). In addition, the catalytic constant (*K*_cat_) of the wild type HIV-1 PR (*K*_cat_ = 0.79 ± 0.11 s^−1^) was almost 2-fold higher than the mutants (*K*_cat_ for MUT-1 = 0.48 ± 0.10 _S_^−1^, *K*_cat_ for MUT-2 = 0.44 ± 0.01 _S_^−1^, and *K*_cat_ for MUT-3 = 0.39 ± 0.01 _S_^−1^). The higher *K*_m_ and lower *K*_cat_ values for the mutant HIV-1 PR resulted in a lower catalytic efficiency (*K*_cat_/*K*_m_) than the WT HIV-1 PR. The catalytic efficiency (*K*_cat_/*K*_m_) of the WT HIV-1 PR (0.021 ± 0.003 _S_^−1^µM^−1^) was markedly higher than in the mutant variants (*K*_m_/*K*_cat_ for MUT-1 = 0.0071 ± 0.001 _S_^−1^µM^−1^, *K*_m_/*K*_cat_ for MUT-2 = 0.0065 ± 0.001 _S_^−1^µM^−1^, and *K*_m_/*K*_cat_ for MUT-3 = 0.0055 ± 0.001 _S_^−1^µM^−1^).

### 3.3. Inhibition of Wild Type HIV-1 PR and Mutants by LPV and DRV

The inhibition data for the WT and mutant HIV-1 PR by LPV and DRV are shown in Table 1 and Figure 3E–H. Both LPV and DRV effectively inhibited the wild type and mutant proteases. However, it was observed that DRV (*K*_i_ = 1.58 ± 0.11 nM) was more potent than LPV (*K*_i_ = 2.13 ± 0.23 nM) in inhibiting the activity of wild type protease. Both the drugs showed higher *K*_i_ values for mutants as compared to the wild type. The *K*_i_ values for the inhibition of the mutants by LPV (MUT-1 = 46.50 ± 0.14 nM, MUT-2 = 52.63 ± 0.65 nM, and MUT-3 = 76.26 ± 0.09 nM) were about 6–8-fold higher than the *K*_i_ value for DRV (MUT-1 = 5.53 ± 0.09 nM, MUT-2 = 7.80 ± 0.71 nM, and MUT-3 = 11.53 ± 1.09 nM). 

### 3.4. Fluorescence Spectroscopy

**Figure 4:**
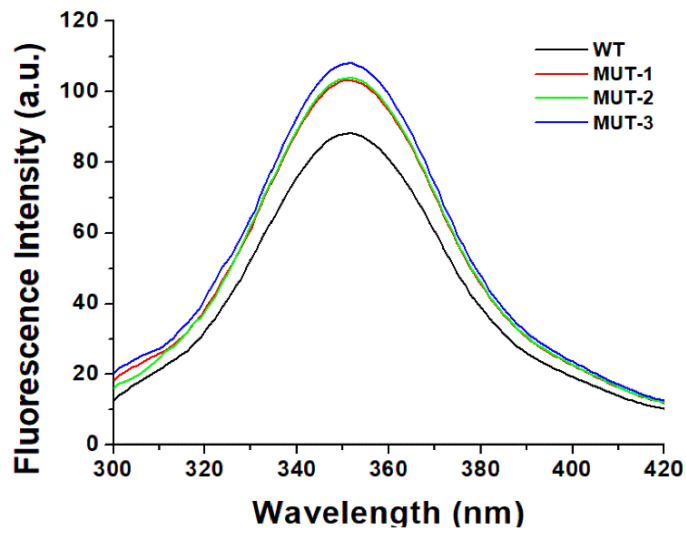
Intrinsic tryptophan fluorescent graph for wild type and mutant HIV-1 PR variants.

**Figure 5:**
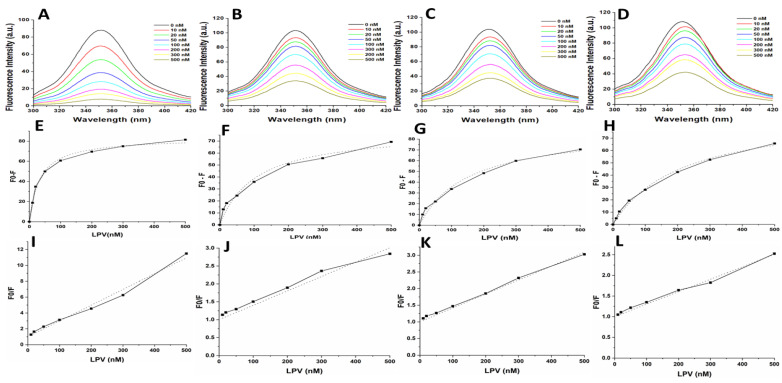
(**A**–**D**) Intrinsic tryptophan fluorescence quenching using LPV for the WT, MUT-1, MUT-2, and MUT-3 respectively. (**E**–**H**) Change in intrinsic tryptophan fluorescence for determination of *K*_i_ using LPV for the WT, MUT-1, MUT-2, and MUT-3 respectively. (**I**–**L**) Stern–Volmer plot to determine the quenching constants (*K*_sv_) using LPV for the WT, MUT-1, MUT-2, and MUT-3 respectively.

**Figure 6:**
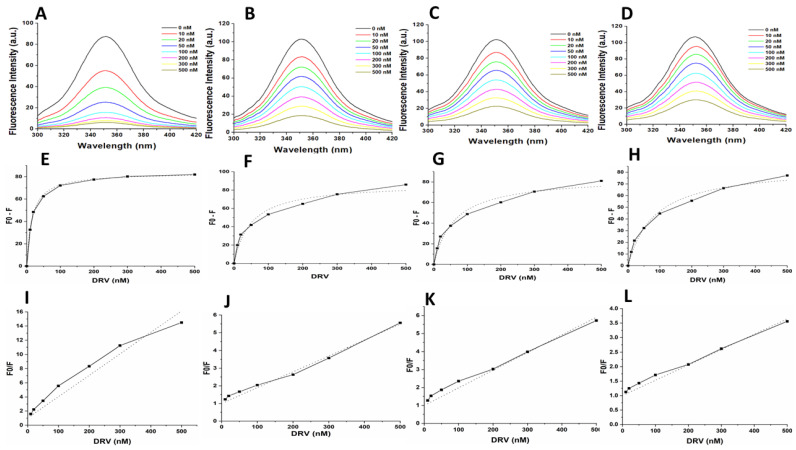
(**A**–**D**) Intrinsic tryptophan fluorescence quenching using DRV for the WT, MUT-1, MUT-2, and MUT-3 respectively. (**E**–**H**) Change in intrinsic tryptophan fluorescence for determination of *K*_i_ using DRV for the WT, MUT-1, MUT-2, and MUT-3 respectively. (**I**–**L**) Stern–Volmer plot to determine the quenching constants (*K*_sv_) using DRV for the WT, MUT-1, MUT-2, and MUT-3 respectively.

Fluorescence spectroscopy was used to investigate secondary and tertiary conformational changes induced in HIV-1 PR due to the binding of the inhibitors LPV and DRV. The WT and mutant HIV-1 PR variants exhibited maximal fluorescence emission spectra (λ_max_) at 351 nm due to the radiative decay associated with the π–π* transition state of HIV-1 PR Trp residues, indicating that the tryptophan residue environment is hydrophilic in nature (Figure 4). The intrinsic Trp fluorescence intensity of the mutant HIV-1 PR variants was increased (14–26%) relative to that of the wild type. A concentration-dependent tryptophanyl fluorescence quenching was observed upon titration of HIV-1 PR with the inhibitors LPV and DRV (Figure 5A–D and Figure 6A–D). There was no red or blue shift in the λ_max_ observed upon increase in the concentration of either of the inhibitors, indicating that the enzymes’ secondary structures remain intact [40]. In addition, the gradual decrease in fluorescence intensity of the HIV-1 PR variants observed as a result of the increase in the concentration of LPV and DRV (Figure 4 and Figure 5A–D) is due to the formation of the enzyme–inhibitor complex [56].

The calculated *K*_i_ values for the WT and mutant PRs from the obtained florescence data are shown in Table 2 and Figure 5 and Figure 6E–H. The WT HIV-1 PR *K*_i_ value (17.25 nM) for LPV was found to be about 3–7-fold lower than in the mutant HIV-1 PR variants (MUT-1 = 54.74 nM, MUT-2 = 79.47 nM, and MUT-3 = 113.16 nM). The WT HIV-1 PR *K*_i_ value (8.12 nM) for DRV was approximately 3–6-fold lower than the mutants (MUT-1 = 26.34 nM, MUT-2 = 32.85 nM, and MUT-3 = 44.70 nM). The fluorescence data obtained was also analyzed to calculate the Stern–Volmer constant (*K*_sv_) by plotting linear Stern–Volmer plots (F_0_/F vs. inhibitor concentrations) (Figure 5 and Figure 6I–L) [57]. It was observed that the *K*_sv_ value (Table 2) for the interaction of LPV with the WT (0.02 nM^−1^) was higher than the mutant HIV-1 PRs (MUT-1 = 0.004 nM^−1^, MUT-2 = 0.004 nM^−1^, and MUT-3 = 0.003 nM^−1^) and similar results were observed for DRV interaction with the WT (0.03 nM) and mutants (0.01 nM^−1^, 0.009 nM^−1^, and 0.005 nM^−1^ for MUT-1, MUT-2, and MUT-3, respectively (Table 2). 

### 3.5. Molecular Dynamic Simulation

#### 3.5.1. Stability of WT, MUT-1, MUT-2, and MUT-3-Inhibitor Complex

**Figure 7:**
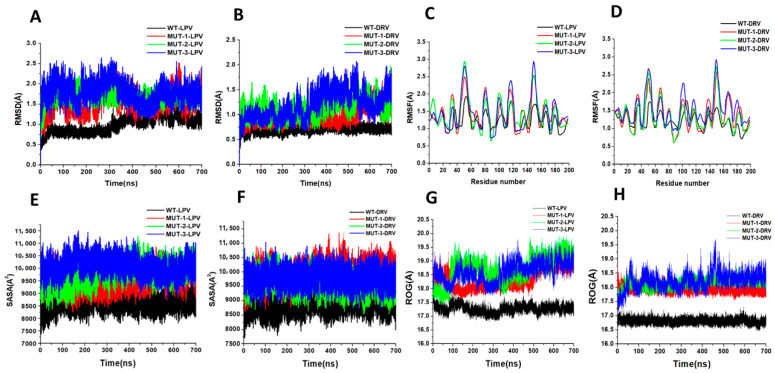
(**A**) Root mean square deviation (RMSD) for WT and mutant HIV-1 PR variants bound to LPV. (**B**) RMSD for WT and mutant HIV-1 PR variants bound to DRV. (**C**) RMSF Figure 1. PR variants bound to LPV. (**D**) RMSF for WT and mutant HIV-1 PR variants bound to DRV. (**E**) Solvent-accessible surface area (SASA) for WT and mutant HIV-1 PR variants bound to LPV. (**F**) SASA for WT and mutant HIV-1 PR variants bound to DRV. (**G**) Radius of gyration (ROG) for WT and mutant HIV-1 PR variants bound to LPV. (**H**) ROG for WT and mutant HIV-1 PR variants bound to DRV.

The dynamic stability of the MD simulation was evaluated using the root mean square deviation (RMSD) of backbone carbon atoms of the different HIV-1 PR variants in complex with LPV and DRV (Figure 7A,B). The lower the RMSD, the more stable the protein complex. The RMSD for the HIV-1 PR-DRV complexes was relatively stable compared to the HIV-1 PR-LPV complexes. The fluctuation in HIV-1 PR amino acid residues as they interact with LPV and DRV throughout the trajectory was monitored using root mean square fluctuation (RMSF) of Cα atoms (Figure 7C,D). This gives an insight into the structural flexibility of the different regions of the HIV-1 PR variants. Interestingly, marked fluctuation in the flap residues (residues 45–55/45′–55′) of the mutant HIV-1 PR variants in complex with LPV and DRV was observed compared to the wild type. A similar fluctuation was seen around the 80′s loop in the mutant HIV-1 PR-LPV complexes.

#### 3.5.2. Solvent Exposure and Radius of Gyration of WT and Mutant HIV-1 PRs

This study determined the solvent-accessible surface area (SASA) of WT and different mutant HIV-1 PRs to LPV and DRV to gain clarity about the hydrophobic core compactness of HIV-1 PR-LPV and DRV complexes. In the HIV-1 PR-LPV complexes (Figure 7E), the SASA for the WT PR (8620.53 A^2^) was lower compared to the SASA of the different mutants (9562.60, 9796.39, and 10,090.94 A^2^ for MUT-1, MUT-2, and MUT-3 respectively). Similarly, the mean SASA for the WT-DRV complex (Figure 7F) (8899.49 A^2^) was lower than the different mutants (9961.57, 9605.59, and 9786.64 A^2^ for MUT-1, MUT-2, and MUT-3, respectively). This high SASA in the mutant LPV and DRV HIV-1 PR complexes may be due to a destabilization of the hydrophobic core. Further confirmation of the instability and gain in flexibility of the mutant HIV-1 PR variants was obtained from the ROG. There was an increase in the ROG for the mutants compared to the WT-LPV and DRV complex (Figure 7G,H). The mean ROG value of the WT HIV-1 PR complexed to LPV (Figure 7G) was found to be 17.28 ± 0.14 Å, and 18.39 ± 0.38, 18.73 ± 0.45, and 18.64 ± 0.34 Å, respectively, for MUT-1, MUT-2, and MUT-3. In the HIV-1 PR-DRV complexes (Figure 7H), the mean ROG value for the WT was found to be 16.82 ± 0.09Å, and 17.94 ± 0.1, 18.16 ± 0.15, and 18.25 ± 0.29 Å, respectively, for MUT-1, MUT-2, and MUT-3.

#### 3.5.3. HIV-1 PR Flap Dynamics 

##### Distance between Active Site Residue to Flap Tip Residue

**Figure 8:**
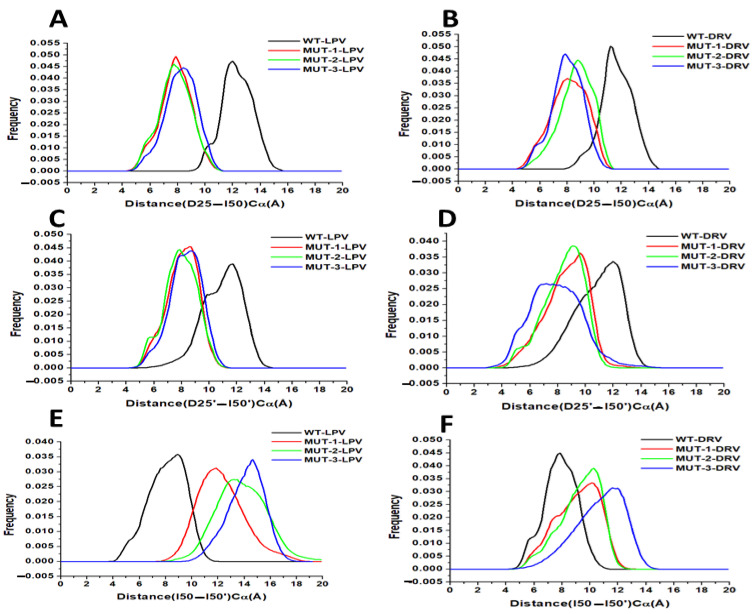
(**A**) Histogram distribution of D25-I50 distance for WT and mutant HIV-1 PR variants interaction with LPV. (**B**) Histogram distribution of D25-I50 distance for WT and mutant HIV-1 PR variants interaction with DRV. (**C**) Histogram distribution of D25′-I50′ distance for WT and mutant HIV-1 PR variants interaction with LPV. (**D**) Histogram distribution of D25′-I50′ distance for WT and mutant HIV-1 PR variants interaction with DRV. (**E**) Histogram distribution of I50-I50′ distance for WT and mutant HIV-1 PR variants interaction with LPV. (**F**) Histogram distribution of I50-I50′ distance for WT and mutant HIV-1 PR variants interaction with DRV.

The distance frequency distribution between the Cα D25–I50 (chain A) in the WT and mutant HIV-1 PR-LPV complexes is plotted in Figure 8A, and the highest peak values were 12.01, 7.82, 7.73, and 8.40 Å for the WT, MUT-1, MUT-2, and MUT-3, respectively. The distance frequency distribution between the Cα D25–I50 (chain A) in the HIV-1 PR-DRV complexes is plotted in Figure 8B, and the highest peak values were 11.33, 7.86, 8.0, and 8.8 Å for the WT, MUT-1, MUT-2, and MUT-3, respectively. The distance between active site to flap tip distance for chain B (D25′–I50′) for the HIV-1 PR-LPV complexes (Figure 8C) was 11.60, 8.67, 7.87, and 8.53 Å for WT, MUT-1, MUT-2, and MUT-3, respectively. The frequency distribution of the distance between Cα D25′ and I50′ (chain B) for the HIV-1 PR-DRV complexes are shown in Figure 8D. These values were 12.13, 9.73, 9.20, and 8.13 Å for WT, MUT-1, MUT-2, and MUT-3. The results obtained showed the Cα D25–I50 and Cα D25′–I50′ distance distribution for the mutant HIV-1 PRs were significantly narrower than in the WT for both the DRV and LPV WT complexes, which is an indication that the presence of these mutations in HIV-1 PR impact the binding of LPV and DRV by causing a compression of the hydrophobic cavity, thus reducing the active site volume of the mutant HIV-1 PRs.

##### Flap Tip to Flap Tip Distance

This study explored the relative motion of the flap tips; this is the distance between Cα I50–I50′. The distance frequency distribution plot between flap tips in the HIV-1 PR LPV complexes (Figure 8E) was 8.93, 11.73, 13.33, and 14.53 Å for WT, MUT-1, MUT-2, and MUT-3, respectively, and 7.87, 10.13, 10.40, and 11.73 Å for WT, MUT-1, MUT-2, and MUT-3 respectively for the HIV-1 PR DRV complexes (Figure 8F). The narrower flap tip to flap tip distance seen between the WT HIV-1 PR-LPV and DRV complexes suggests that these inhibitors bind tightly. The large distances between flap tips seen in the mutant PRs indicate open movements in the flap tips and loose binding of these PIs to the mutant HIV-1 PRs. The observed decrease in the Cα D25–I50 and Cα D25′–I50′ distance and increase in Cα I50–I50′ in MUT-1, MUT-2, and MUT-3 DRV/LPV complexes agreed with the earlier observation showing high RMSF around the flap residues (residues 45–55/45′–55′) in the mutant HIV-1 PR-LPV and DRV complexes.

#### 3.5.4. Structural Comparison of WT and Mutant HIV-1 PR 

**Figure 9:**
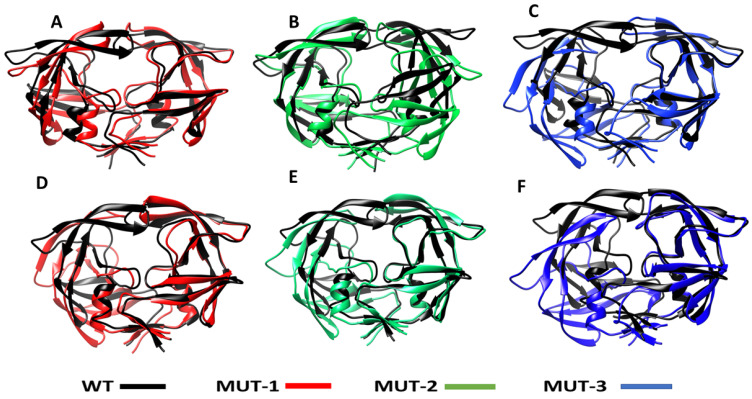
Superimposed structure of WT and the different mutants LPV and DRV complexes. (**A**) Superimposed structure of the WT-LPV and MUT-1-LPV complex. (**B**) Superimposed structure of the WT-LPV and MUT-2-LPV complex. (**C**) Superimposed structure of the WT-LPV and MUT-3-LPV complex. (**D**) Superimposed structure of the WT-DRV and MUT-1-DRV complex. (**E**) Superimposed structure of the WT-DRV and MUT-2-DRV complex. (**F**) Superimposed structure of the WT-DRV and MUT-3-DRV complex.

Structural comparison of the structures of HIV-1 PR WT and mutants, when bound to LPV and DRV, is shown in Figure 9 (generated during the last 20 ns of the simulation). It can be observed that the flaps of the mutant variants (MUT-1, MUT-2, and MUT-3) when bound to LPV and DRV was in an open conformation compared to the closed conformation in the wild type. In addition, the overall structures of the mutant versus the wild seem altered, which may be due to the impact of the mutations, causing a reorganization of the HIV-1 PR structure. This finding may be associated with the fluctuation in the flap region seen in the RMSF and the high RMSD observed in the mutant HIV-1 PR variants compared to the wild type.

#### 3.5.5. HIV-1 PR Binding Profile Calculated from MMGBSA 

To determine the impact of the mutations on the HIV-1 PR binding landscape, binding free energy of LPV and DRV to the WT and mutant HIV-1 PRs was calculated using the MM-GBSA method (Table 3). The binding energies of LPV (−43.25 kcal/mol) and DRV (−48.19 kcal/mol) to WT were high compared to the binding energies of these inhibitors to the different mutant PRs. The binding energies of DRV to the different mutant PRs (−31.51, −24.43, and −21.58 kcal/mol for MUT-1, MUT-2, and MUT-3, respectively) were found to be higher than the binding energies of LPV to mutants (−25.39, −26.67, and −20.28 kcal/mol for MUT-1, MUT-2, and MUT-3, respectively). The increased binding energy in the HIV-1 PR-DRV complexes may be attributed to the relatively low solvation energy and increased electrostatic interaction compared to the HIV-1 PR-LPV complexes.

#### 3.5.6. Hydrogen Bond Interaction Analysis

**Figure 10:**
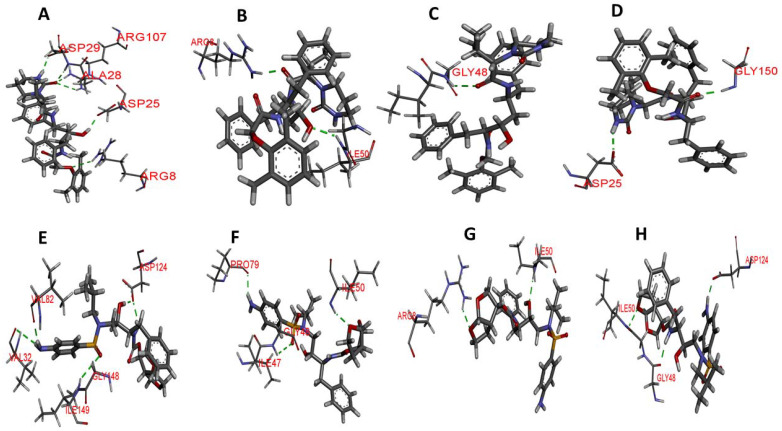
Hydrogen bond interaction of wild type and mutant HIV-1 PR variants with LPV and DRV. The green broken lines represent the hydrogen bond between amino acid residues of HIV-1 PR and the inhibitors and (**A**–**D**) hydrogen bond interaction of WT, MUT-1, MUT-2, and MUT-3 with LPV, respectively. (**E**–**H**) Hydrogen bond interaction of WT, MUT-1, MUT-2, and MUT-3 with DRV, respectively.

To further determine the level of interaction and stability between HIV-1 PR-LPV and DRV complexes, hydrogen bond analysis of the snapshots from the last 20 ns was analyzed using discovery studio. Table 4 and Figure 10 shows the key hydrogen bond interactions that were found between LPV and DRV complexes of the WT, MUT-1, MUT-2, and MUT-3 PRs, respectively. The hydrogen bond distances are also presented in Table 4. In the HIV-1 PR-LPV complexes, it was observed that the LPV formed hydrogen bonds with residues ARG 8, ARG 107, ASP 25, and ASP 29 in the wild type. Most of these bonds were lost in the mutant HIV-1 PR variants. LPV formed hydrogen bonds with ARG 8 and ILE 50 in the MUT-1-LPV complex, GLY 48 in the MUT-2-LPV complex, and ASP 25 and GLY 150 in the MUT-3-LPV complex. In the WT-DRV complexes, DRV formed hydrogen bonds with residues ASP 124, VAL 32, VAL 82, and ILE 149. In the mutant HIV-1 PR-DRV complexes, fewer hydrogen bonds were formed between the drug and the protein. DRV formed hydrogen bonds with GLY 48, ILE 50, and PRO 79 in the MUT-1-DRV complex, ARG 8 and ILE 50 in the MUT-2-DRV complex, and ASP 124, GLY 48, and ILE 50 in the MUT-3-DRV complex. 

## 4. Discussion

This study describes the biochemical and structural characteristics of multidrug-resistant HIV-1 PR, cloned from clinical isolates obtained from patients at the point of switching from LPV to DRV-based regimen. This study used a prokaryotic host (*Escherichia coli*) expression system to express and characterize the HIV-1 PR variants. The eukaryotic host expression systems (yeast and mammalian cells) have been used basically to study HIV-1 PR-PI drug susceptibility. However, the *Escherichia coli* expression system has been extensively used for functional and structural characterization of HIV-1 PR (see review [58]). We found that the combination of the mutations harbored by the mutant HIV-1 PR variants significantly impacted enzyme catalytic activity. The *K*_cat_/*K*_m_ progressively decreased with an increase in the number of drug-resistant mutations. Despite the reduced affinity and catalytic efficiency, the mutants could still cleave the substrate. This can be explained by an earlier study by Prabu-Jeyabalan et al.; these authors showed that the interaction between the active site residues and the substrate is relatively conserved even in the presence of mutations, thus favoring substrate cleavage, but the binding landscape of HIV-1 PIs is altered [59].

The inhibitory constants (*K*_i_) show that the mutations severely affected the *K*_i_ for both LPV and DRV (Table 1, Figure 3E–L). In contrast to the progressive decrease observed in the *K*_cat_/*K*_m_, there was a marked increase in the *K*_i_ associated with an increase in the number of mutations for LPV and DRV. This interdependence of the *K*_i_ value on the *K*_cat_/*K*_m_ may play a role in the altered recognition of HIV-1 PIs and their binding to the active site. The results of similar studies also show a marked decrease in the catalytic efficiency of the multidrug-resistant HIV-1 PR variants relative to an increase in the *K*_i_ of HIV-1 PIs [60,61,62]. This indicates the trade-off of catalytic efficiency for decreased inhibitory activity of HIV-1 PIs may be a means of responding to drug selection pressure, which may confer an evolutionary advantage on the mutated HIV-1 PR [63]. The error prone nature of HIV-1 replication places HIV-1 under strong selective pressure resulting in the rapid accumulation of drug resistance mutations [64]. Under selective pressures proteins evolve to become tolerant to mutations and remain catalytically viable despite the impact the mutations on their catalytic efficiency [64,65]. The catalytic efficiency of the mutant HIV-1 PR variants in this study was between 26 and 34% of the WT HIV-1 PR. This result agrees with the findings of similar studies [62,66]. A study has shown that mutant HIV-1 PR variants with catalytic efficiency as low as 7% of the WT HIV-1 PR are catalytically viable [62].

The results of this study indicate that both LPV and DRV successfully inhibited the mutant HIV-1 PR variants. However, a lower dose of DRV was needed to inhibit the HIV-1 PR variants showing the superiority of DRV over LPV. In addition, this finding supports the switch to a DRV-based regimen after LPV failure. The high resistance to LPV and intermediate resistance to DRV showed an association with the decreased *K*_ca_t/*K*_m_ and increased *K*_i_ in the mutant HIV-1 PR variants. As has been described in a previous study, the lower resistance to DRV compared to LPV may be due to the improved design of DRV, which allows it to fit tightly in the active site, coupled with the increased hydrogen bond interaction with the HIV-1 PR backbone, conferring on it a high binding affinity for the active site [67]. In a clinical trial that evaluated the long-term effectiveness of ART, the results showed that a DRV-based regimen was superior to an LPV-based regimen in both ART-naive and treatment-experienced patients. The chances of virological failure were lower for DRV compared to LPV when HIV-1 PI therapy was initiated either as a salvage regimen or a switching strategy in treatment-experienced patients [68]. Another study also showed that the use of low dose DRV boosted with ritonavir (RTV) is an efficient switch option to suppress virological failure in patients failing LPV based treatment [69]. In the current study, though DRV better inhibited the HIV-1 PR variants than LPV, the relative resistance (Table 1) to LPV and DRV followed a similar trend. This may be associated with the similarities in the chemical and structural signatures of the inhibitors, which are designed to mimic the HIV-1 PR substrate transition state; thus, the evolution of resistance to HIV-1 PI usually may follow a similar pattern [70]. In addition, most of the mutations harbored by the mutant HIV-1 PR variants in this study are LPV resistance mutations except L33F (in MUT-2) and L76V (in MUT-2 and 3). This may have caused the pattern of resistance to LPV and DRV to be similar, as the presence of more DRV associated mutations may have changed the dynamics or pattern of resistance to DRV seen in this study.

The *K*_i_ values obtained in this study followed a similar pattern to those obtained from other studies. The *K*_i_ values in this study for the wild type South African HIV-1 subtype C PR (*K*_i_ = 2.13 ± 0.23 nM for LPV and *K*_i_ = 1.58 ± 0.11 nM for DRV) is close to that reported in a previous study (*K*_i_ = 2.1 ± 0.2 nM for LPV and *K*_i_ = 1.4 ± 0.2 nM for DRV) [71]. However, there is no other study reporting the kinetic properties of South African HIV-1 subtype C PR harboring multidrug-resistant mutations and their interaction with LPV and DRV. Extensive research has been done on the kinetic characterization of HIV-1 subtype B PR, and the *K*_i_ values of the wild type and different multidrug-resistant forms are known. The LPV *K*_i_ value of the wild-type subtype B HIV-1 PR was approximately 0.02 nM. The multidrug-resistant forms harboring numerous major and minor mutations showed *K*_i_ values of 0.44–260 nM [60,72,73]. The DRV *K*_i_ value for the wild type subtype B was around 0.005 nM and the multidrug-resistant forms harboring numerous major and minor mutations with *K*_i_ values ranging from 1.8 to 41 nM [74]. Variation in the affinity of subtypes B and C HIV-1 PR for PIs has been attributed to the signature polymorphisms in HIV-1 subtype C. The in vitro characterization of HIV-1 PR has shown that HIV-1 PIs better inhibit the wild type and mutant forms of HIV-1 subtype B PR compared to HIV-1 PR variants from the subtype C [36,37]. In another study, patients infected with HIV-1 subtype C receiving ritonavir-boosted regimens developed secondary virological failure within a shorter time compared to patients infected with HIV-1subtype B [75]. This current study is essential as it addresses the paucity of data regarding the biochemical interaction of multidrug-resistant South African HIV-1 subtype C PR variants with LPV and DRV, commonly used in the formulation of salvage regimens.

The findings discussed thus far highlights the broad impact of multidrug-resistant mutations on the molecular interaction and binding landscape of HIV-1 PR variants with the substrate, LPV, and DRV. Though this presents a critical picture of the evolution of resistance to LPV and DRV, it does not address other key factors at the molecular level that may shape the interaction and binding landscape of LPV and DRV with the HIV-1 PR variants. As a result, we explored the use of intrinsic Trp fluorescence to assess conformational changes in the three-dimensional structure of HIV-1 PR variants associated with these mutations and the binding of LPV and DRV [40,76]. The increased intrinsic fluorescence emission spectra in the mutant HIV-1 PR variants compared to the wild type in the absence of an inhibitor (Figure 4) indicates that the Trp residues in the mutant HIV-1 PR variants are more solvent-exposed. This finding highlights the impact of the drug-resistant mutations on the tertiary structural changes in the mutant HIV-1 PR variants [4]. The more solvent-exposed the HIV-1 PR dimer is, the more unstable the enzyme–inhibitor complex becomes [23]. These changes in the intrinsic HIV-1 PR conformational flexibility associated with resistance mutations from LPV-induced drug pressure may cause permanent changes in the HIV-1 PR structure leading to an unfavorable binding landscape to DRV-based regimen after therapy switch. Research has shown that the resistance mutations selected during ART introduce conformational changes to the HIV-1 PR structure, leading to the evolution of HIV-1 PR variants resistant to several HIV-1 PIs since most of these inhibitors are similar in their chemical nature [77,78,79]. While the changes in HIV-1 PR structural conformation selected during a particular PI therapy has been shown to cause cross-resistance to several PIs, it has not been demonstrated for the switch from LPV to DRV therapy using HIV-1 PR from clinical isolates.

Further supporting the suggestion that the tertiary structural changes observed in the mutant HIV-1 PR variants are a product of the drug-resistant mutations harbored is the results obtained from the calculation of the *K*_sv_ value. The low *K*_sv_ values from the interaction of the mutant HIV-1 PR variants with LPV and DRV compared to the WT (Table 2) indicate that the structures of the mutant HIV-1 PR variants are in a more open state rather than in a closed conformational state. This may have emerged from structural reorganization caused by the mutations harbored, resulting in a less tight binding and accessibility of Trp residues, thus the resultant low quenching capacity of the inhibitors observed with the mutant HIV-1 PR variants. This finding is corroborated by the highly open flap and open protein conformation observed in the molecular dynamic simulation (Figure 8 and Figure 9). The *K*_sv_ value also throws more light on the efficiency of LPV and DRV to inhibit the HIV-1 PR variants. The overall observation from the *K*_sv_ value shows that DRV had a relatively higher quenching capacity than the LPV. This may be due to the tight-fitting and high binding affinity of DRV to the active site compared to LPV, as mentioned earlier [67]. This further confirms the superiority of DRV to LPV.

From the preceding discussion, it can be inferred that increased resistance to LPV, intermediate resistance to DRV, decreased catalytic efficiency, and altered tertiary structure of HIV-1 PR observed in this study evolved due to the mutations selected from LPV-induced drug pressure. Earlier studies have shown that intrinsic changes in the HIV-1 PR conformation associated with the accumulation of drug-resistant mutations may cause geometric alteration in the HIV-1 PR structure affecting the active site and other domains [61,77]. These intrinsic changes may cause sensitization and cross-resistance to HIV-1 PIs by altering the molecular interaction of the former with the protein during binding [16,77]. The evolution of these characteristics in proteins is often associated with the loss of protein stability [80]. The alteration in the HIV-1 PR tertiary structure, observed from fluorescent spectroscopy results, agreed with the root mean square deviation (RMSD), root mean square fluctuation (RMSF), the radius of gyration (ROG), and solvent accessibility area (SASA) results (Figure 7A–H) obtained from the molecular dynamic simulation.

The increased fluctuation in the RMSF (Figure 7C,D) values, the unstable RMSD (Figure 7A,B) of the mutant HIV-1 PR LPV, and the DRV complexes confirm the intrinsic conformational instability induced by these drug-resistant mutations. This signifies a disruption in protein structure, leading to a loss of compactness affecting LPV and DRV binding. This finding can also be associated with the high SASA (Figure 7E,F) and ROG (Figure 7G,H) in the mutant HIV-1 PR variants when bound to LPV and DRV. These results agreed with the findings of a similar study by Chetty et al., which investigated the impact of multidrug-resistant mutations on the HIV-1 subtype B PR resistance profile and molecular dynamic characteristics. The authors of this study showed that the accumulation of multidrug-resistant mutations causes inherent changes in the structures of HIV-1 PR, resulting in HIV-1 PR variants with increased conformational flexibility and an open conformation. Thus, causing an increase in the rate of dissociation of PIs bound to the active site [23]. The increased SASA and ROG of the mutant HIV-1 PR, when bound to LPV and DRV in this study, indicate a loss of hydrophobic core compactness, and this has been shown to modulate the activity of HIV-1 PR and the drug binding landscape [81].

Alteration in the HIV-1 PR hydrophobic core has been proposed as a mechanism by which mutations distant from the active site cause drug resistance [81]. These mutations do not interact directly with the inhibitor but cause an alteration in the balance between substrate recognition and inhibitor binding in a way that favors interaction with the natural substrate and alters the drug binding landscape [81]. The L76V mutation found in MUT-2 and MUT-3 in this study has been shown previously to confer resistance to several HIV-1 PIs through the local rearrangement of the hydrophobic core [82]. It is also associated with decreased stability of the HIV-1 PR dimer [82] and emerges during treatment with an LPV-based regimen [27]. The L76V is also commonly seen in highly mutated HIV-1 PR variants resistant to DRV [83]. The L90M observed in MUT-3 is another mutation associated with the alteration of the HIV-1 PR hydrophobic core flexibility and decreased dimer stability. Though this mutation does not make contact with PIs in the active site, it can cause cross-resistance to most PIs except DRV and tipranavir (TPV) [81,83,84]. The L90M mutation is not commonly seen in HIV-1 subtype C PR, this mutation may have evolved from previous exposure of the patients to PIs like nelfinavir (NFV) and saquinavir (SQV) before treatment with the LPV containing regimen [85]. A comparative analysis of the genotypic variation in the HIV-1 PR from subtype B and C from laboratory-generated sequences and publicly available database showed the L90M occur more in HIV-1 subtype B PR than HIV-1 PR variants from subtype C [86].

The hydrophobic interactions between the hydrophobic amino acid residues in the core are vital in maintaining the conformational flexibility of all HIV-1 PR regions, including the flap dynamics. Thus, the hydrophobic core state also modulates HIV-1 PR flap behavior when binding to HIV-1 PIs [81,87]. This, therefore, shows that the open and increased flap flexibility (Figure 8A–F) in the mutant HIV-1 PR variant may be a product of the distorted hydrophobic core, as shown by the increased SASA in addition to the presence of the flap mutations M46I and I54V present in all the mutants studied. Flap flexibility contributes to ligand stability in the HIV-1 PR active site and influences the binding affinity of HIV-1 PR to PIs [88,89,90]. Therefore, the increased flap flexibility of the mutant HIV-1 PR variants in this study contributed to the high-level resistance to LPV and intermediate resistance DRV. The M46I and I54V flap mutations found in the mutants in this study have been shown in a previous study to be associated with decreased binding affinity of HIV-1 PR to PIs, even though they are not active site mutations [91]. These mutations, in the presence of active site mutations, like V82A and I84V, cause high-level cross-resistance to several HIV-1 PIs [61]. In addition, the F53L mutation in MUT-3, though a minor mutation in the flap, is associated with the loss of the hydrophobic bond interaction between phenylalanine’s side chain at position 53 of chain A, with isoleucine at position 50 on the second PR subunit (chain B). Thus, resulting in a wider gap between the two flaps, keeping the HIV-1 PR variants in an open conformation [92]. Enzyme kinetics models have been used to show that HIV-1 PR mutations that distort the balance between a closed and open conformation to favor an open conformation keep the enzyme catalytically active regardless of a PI in the active site. This confers high-level resistance on HIV-1 PR to PIs [93].

Analysis of the molecular interaction of the HIV-1 PR variants showed that the reduced capacity of the LPV and DRV (compared to the wild type) emanates from the loss of hydrogen bond contacts (Table 4, Figure 10) between the inhibitors and active site residues. The hydrogen bonds between residues in the HIV-1 PR active site and PIs contribute to the latter’s tight binding. The loss of these interactions promotes the dissociation of inhibitors from the enzyme–inhibitor complex and is related to the level of resistance to HIV-1 PIs [51,94]. The loss in hydrogen bonds and changes induced in HIV-1 PR conformations observed in this study together translate into the low binding energy between the mutant HIV-1 PR variants with LPV and DRV observed (Table 3). The high binding energy in the HIV-1 PR-DRV complexes shows that DRV interacted better with the HIV-1 PR variants, and this may be the reason for the better stability of the HIV-1 PR-DRV complexes. The presence of the V82A mutation in all the HIV-1 PR mutant variants may have contributed significantly to the reduced binding affinity of the mutant HIV-1 PR variants to LPV and DRV. The V82 amino acid residue is located in a region critical to drug and substrate binding as it makes direct contact with both HIV-1 PIs and substrate. The V82A mutation is common in patients failing LPV therapy and emerges from the sustained use of LPV during virological failure [27]. This confers resistance on HIV-1 PR through a structural shift of residues in the 80′s loop [95]. Taken together these findings, from applying a multidimensional approach in studying the evolution of drug resistance to LPV and DRV during ART switch, is critical as it gives an insight into the efficiency of the switch from LPV and DRV, which is a common practice.

## 5. Conclusions

This study’s findings provide mechanistic insight into the link between acquired conformational flexibility, associated with resistance mutations selected during an earlier treatment, and its impact on the outcome of a therapy switch. The HIV-1 PR structural changes associated with mutations that emerge due to drug pressure from LPV treatment during virological failure may shape the DRV binding landscape, affecting the switch to a DRV-based regimen. Though the drug-resistant HIV-1 PR variants showed intermediate resistance to DRV, the latter has proven to be more effective than LPV in inhibiting the wild type and mutant HIV-1 PR variants. The capacity of DRV to be effective against HIV-1 PR with already altered conformations from earlier LPV treatment makes it the drug of choice over LPV. This study provides essential information for the development of future inhibitors to address the impact of the altered binding landscape that evolves from initial treatments to help achieve long-term virological suppression.

## Figures and Tables

**Table 1:** Wild type and mutant HIV-1 PR variants enzyme kinetic parameters (*K*_m_, *K*_cat_) and inhibition constant (K_i_) calculated using the chromogenic substrate.

HIV-1 PR Variants	*K*_m_ (µM)	*K*_cat_ (_S_^−1^)	*K*_cat_/*K*_m_ (_S_^−1^µM^−1^)	LPV		DRV	
				*K*_i_ (nM)	Relative Resistance to LPV	*K*_i_ (nM)	Relative Resistance to DRV
WT	37.49 ± 0.63	0.79 ± 0.11	0.021 ± 0.003	2.13 ± 0.23	1.00	1.58 ± 0.11	1.00
MUT-1 M46I, I54V, V82A, L10F	67.78 ± 1.22	0.48 ± 0.10	0.0071 ± 0.001	46.50 ± 0.14	21.83	5.53 ± 0.09	3.50
MUT-2 M46I, I54V, L76V, V82A, L33F, L10F	67.46 ± 1.48	0.44 ± 0.01	0.0065 ± 0.001	52.63 ± 0.65	24.71	7.80 ± 0.71	4.94
MUT-3 M46I, I54V, L76V, V82A, L90M, F53L	70.59 ± 1.01	0.39 ± 0.01	0.0055 ± 0.001	76.26 ± 0.09	35.80	11.53 ± 1.09	7.30

Relative resistance = *K*_i_ of mutant/*K*_i_ of WT.

**Table 2:** Inhibition constant (K_i_) and Stern–Volmer quenching constants (*K*_sv_) calculated from fluorescence quenching assay.

HIV-1 PR Variants	LPV		DRV	
	*K*_i_ (nM)	*K*_sv_ (nM^−1^)	*K*_i_ (nM)	*K*_sv_ (nM^−1^)
WT	17.25	0.02	8.12	0.030
MUT-1 M46I, I54V, V82A, L10F	54.74	0.004	26.34	0.01
MUT-2 M46I, I54V, L76V, V82A, L10F, L33F	79.47	0.004	32.85	0.009
MUT-3 M46I, I54V, L76V, V82A, L90M, F53L	113.16	0.003	44.70	0.005

**Table 3:** Binding free energies of DRV and LPV to HIV-1 PR variants.

Energy Components	PI	WT-DRV	MUT-1-DRV	MUT-2-DRV	MUT-3-DRV
**ΔE_vdw_**	LPV	−53.35 ± 7.97	−34.67 ± 5.23	−34.46 ± 4.39	−32.96 ± 8.03
	DRV	−51.27 ± 5.51	−36.10 ± 5.66	−32.46 ± 5.23	−33.13 ± 8.31
**ΔE_elec_**	LPV	−26.98 ± 4.65	−25.89 ± 8.30	−24.48 ± 5.06	−25.12 ± 6.21
	DRV	−29.75 ± 8.55	−27.32 ±7.74	−28.91 ± 6.68	−24.38 ± 8.30
**ΔG_gas_**	LPV	−80.33 ± 13.33	−60.56 ± 9.64	−58.94 ± 8.28	−59.08 ± 5.29
	DRV	−81.02 ± 11.63	−65.04 ± 10.43	−61.37 ± 9.80	−57.53 ± 14.07
**ΔG_solv_**	LPV	37.08 ± 8.12	35.17 ± 5.76	35.27 ± 7.93	38.80 ± 6.11
	DRV	31.83 ± 5.68	34.03 ± 7.17	35.92 ± 5.21	35.93 ± 7.96
**ΔG_bind_**	LPV	−43.25 ± 12.30	−25.39 ± 7.76	−23.67 ± 4.49	−20.28 ± 5.53
	DRV	−48.19 ± 9.28	−31.51 ± 6.81	−24.43 ± 6.05	−21.58 ± 7.58

**ΔE_vdw_** = van der Waals free energy; **ΔE_elec_** = electrostatic free energy; **ΔG_gas_ =** gas phase Gibbs free energy; **ΔG_solv_** = solvation energy.

**Table 4:** Key hydrogen bond interactions between active site residues of WT, MUT-1, MUT-2, and MUT-3 with LPV and DRV.

**Hydrogen Bond Interaction**	**Distance (Å)**			
	**WT-LPV**	**MUT-1-LPV**	**MUT-2-LPV**	**MUT-3-LPV**
ARG8:HH21—LPV:O5	2.59	1.93	-	-
ARG107:HH12—LPV:O3	1.77	-	-	-
ARG107:HH22—LPV:O3	2.57	-	-	-
ASP25:OD2—LPV:H27	1.95	-	-	-
ASP25:OD1—LPV:H26	-	-	-	2.08
ASP29:OD1—LPV:H26	2.02	-	-	-
ASP29:H—LPV:OD3	2.04	-	-	-
GLY 48:H—LPV:O2	-	-	2.30	-
ILE50:H—LPV:O1	-	2.20	-	-
GLY 150:H—LPV:O4	-	-	-	1.93
**Hydrogen Bond Interaction**	**Distance (Å)**			
	**WT-DRV**	**MUT-1-DRV**	**MUT-2-DRV**	**MUT-3-DRV**
ARG8:NH1—DRV:O2	-	-	2.12	-
ASP124:OD2—DRV:H14	2.17	-	-	-
ASP124:OD2—DRV:H20	1.69	-	-	-
ASP124:OD2—DRV:H36	-	-	-	1.82
VAL32:O—DRV:H36	2.65	-	-	-
GLY 48:H—DRV:O6	-	2.73	-	-
GLY 48:O—DRV:H14	-	-	-	1.91
ILE50:H—DRV: O2	-	2.06	2.04	2.31
PRO79:O—DRV:H36	-	2.03	-	-
VAL82:O—DRV:H36	1.78	-	-	-
ILE 149:H—DRV:O7	2.95	-	-	-

## Data Availability

Data is contained within the article and the supplementary material; further information can be obtained from the corresponding author.

## Supplementary Materials

The following are available online at https://www.mdpi.com/2218-273X/11/4/489/s1, Figure S1: Unedited gel electrophoresis picture of amplified HIV-1 PR gene, Figure S2: Unedited SDS-PAGE picture of the purified HIV-1 PR gene and MBP tagged HIV-1 PR, Figure S3: Unedited SDS-PAGE picture showing cleavage products after Factor Xa cleavage of HIV-1 PR from the MBP tag, Figure S4: Unedited Western blot picture of the HIV-1 PR variants.
