# Supplementary figures and images for: Acquired HIV-1 Protease Conformational Flexibility Associated with Lopinavir Failure May Shape the Outcome of Darunavir Therapy after Antiretroviral Therapy Switch
Source: Biomolecules. 2021 Mar 24;11(4):489. doi: 10.3390/biom11040489 (PMC8064090; doi:10.3390/biom11040489)

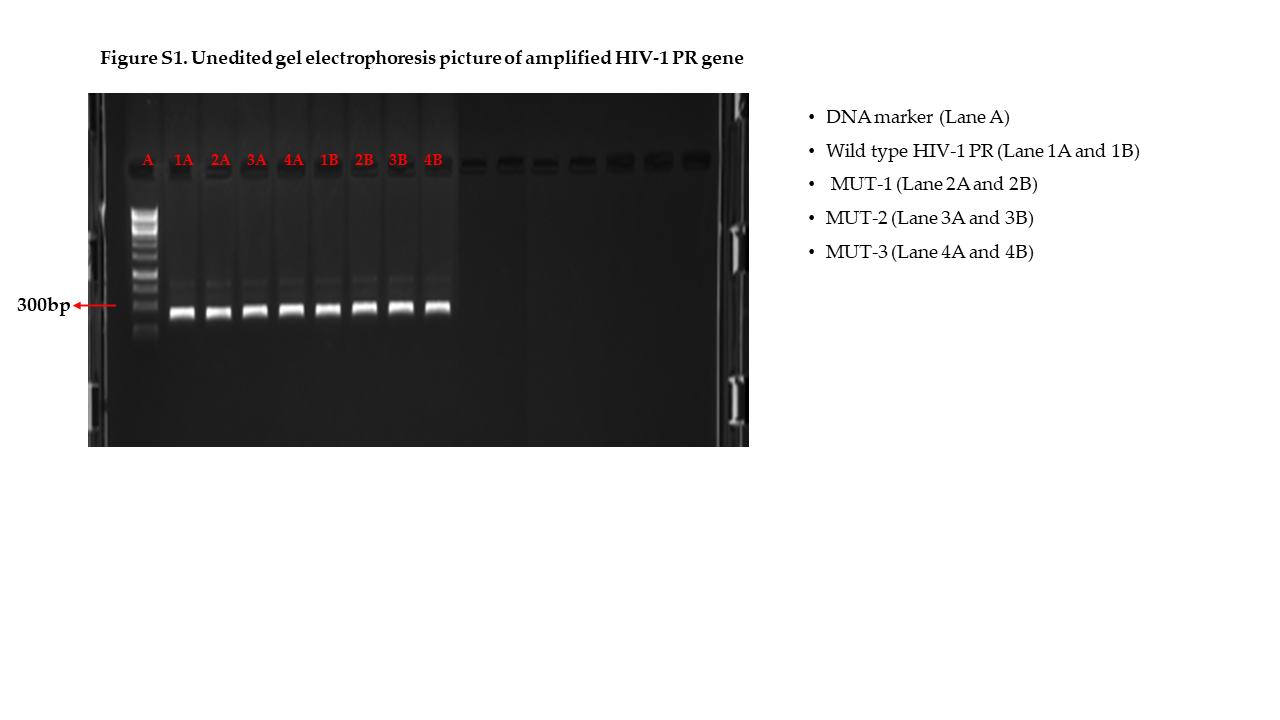

Supplement: Supplementary file 1 [file biomolecules-11-00489-s001.zip › supplemtary tiffs/Slide1.TIF]

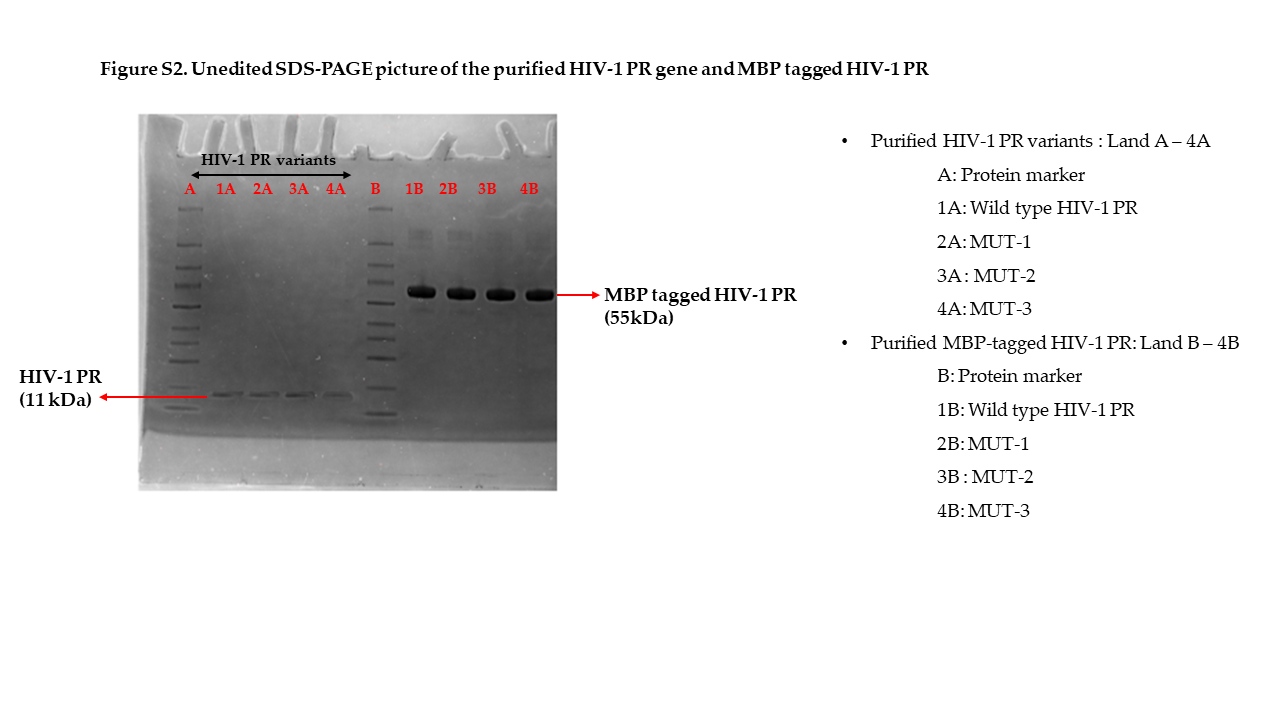

Supplement: Supplementary file 1 [file biomolecules-11-00489-s001.zip › supplemtary tiffs/Slide2.TIF]

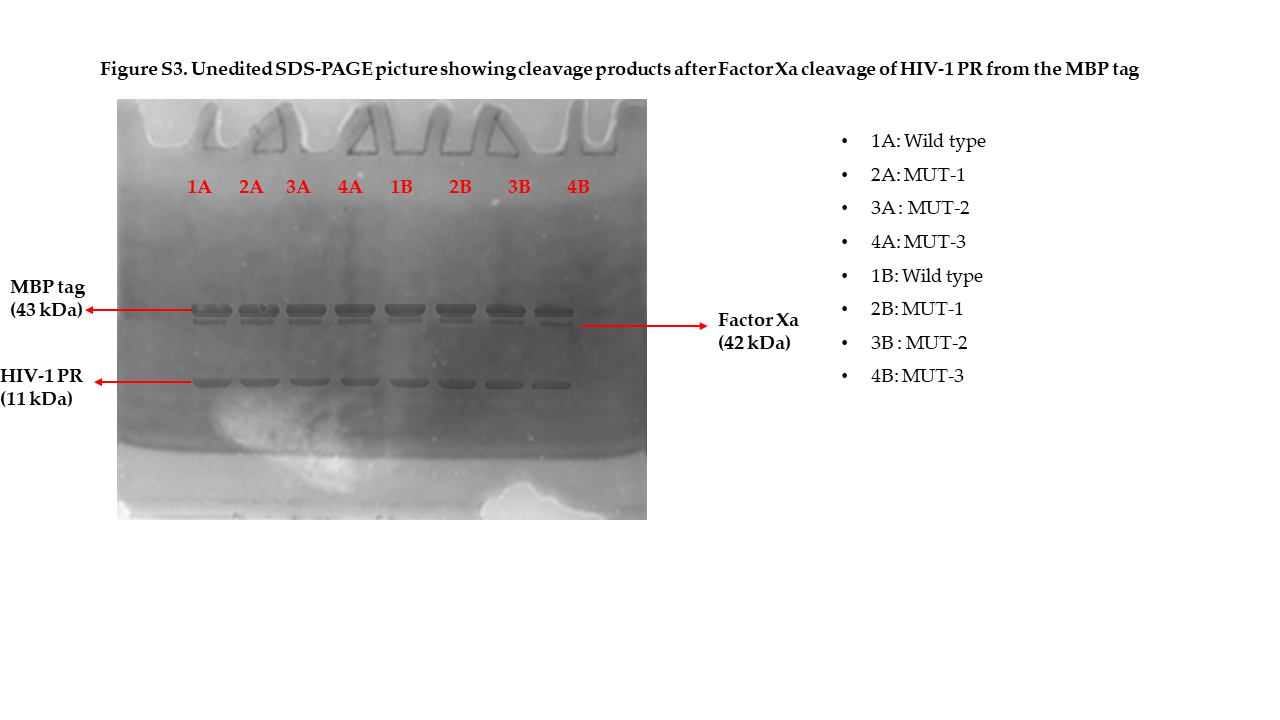

Supplement: Supplementary file 1 [file biomolecules-11-00489-s001.zip › supplemtary tiffs/Slide3.TIF]

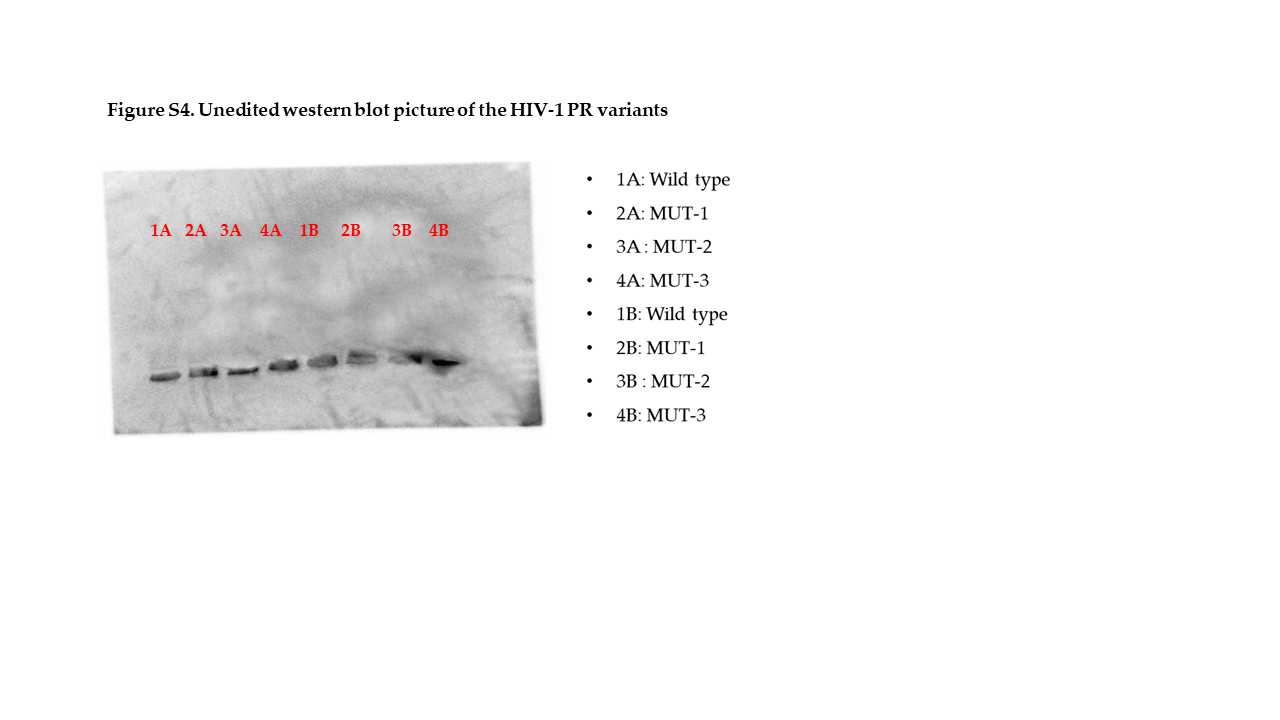

Supplement: Supplementary file 1 [file biomolecules-11-00489-s001.zip › supplemtary tiffs/Slide4.TIF]
